# The Ways in Which Stakeholders Make Decisions About AI and Novel Technologies for the Health Care of Older Adults: Qualitative Interview Study

**DOI:** 10.2196/86148

**Published:** 2026-07-29

**Authors:** Zhang Zhang, Thomas KM Cudjoe, Sato Ashida, Jacqueline Massare, Kacey Chae, Phillip Phan, Peter Abadir, Alicia I Arbaje, Mathias Unberath, Nancy L Schoenborn

**Affiliations:** 1 Department of Health Policy and Management Johns Hopkins Bloomberg School of Public Health Johns Hopkins University Baltimore, NC United States; 2 Bursky School of Public Health Washington University in St. Louis St. Louis, MO United States; 3 Division of Geriatric Medicine and Gerontology, Department of Medicine School of Medicine Johns Hopkins University Baltimore, MD United States; 4 Department of Community and Behavioral Health College of Public Health University of Iowa Iowa City, IA United States; 5 Division of General Internal Medicine, Department of Medicine School of Medicine Johns Hopkins University Baltimore, MD United States; 6 Bariatric and Weight Management, Surgery School of Medicine Wake Forest University Winston Salem, NC United States; 7 Carey Business School Johns Hopkins University Baltimore, MD United States; 8 Johns Hopkins Armstrong Institute for Patient Safety and Quality Johns Hopkins University Baltimore, MD United States; 9 Department of Computer Science Johns Hopkins University Baltimore, MD United States

**Keywords:** artificial intelligence, AI, novel technology, older adults, decision-making, artificial intelligence adoption, AI adoption, usability, technology acceptance model, perceived usefulness

## Abstract

**Background:**

Artificial intelligence (AI) has the potential to improve health among older adults, yet how different stakeholders decide to develop, finance, and adopt AI innovations is not well understood.

**Objective:**

This study aimed to understand the decision-making of different stakeholders regarding AI and technologies for the health care of older adults.

**Methods:**

We conducted semistructured interviews with 15 older adults and care partners, 15 clinicians, 8 health system or insurance leaders, 5 investors, and 6 technology developers. Data were analyzed using thematic content analysis.

**Results:**

All stakeholders considered cost, value, and usability important in adopting AI health technologies but emphasized different aspects of each concept. Older adults and care partners prioritized out-of-pocket costs and ease of use, whereas payers emphasized disease prevalence and implementation feasibility. Developers and investors focused on profitability and scalability, resulting in tension with end users’ priorities. Participant suggestions included problem-driven design, greater stakeholder engagement, public-private partnerships, and educating older adults about AI.

**Conclusions:**

As with prior health-related technology innovations, aligning decisional priorities across stakeholders is critical to motivate impactful AI health technologies for older adults.

## Introduction

AI and novel technologies offer tremendous potential for improving the health of older adults. They are becoming an important focus for technologies that facilitate aging in place, reduce care partner burden, and improve health system efficiency [1-3]. AI, which uses various approaches to simulate human intelligence and behavior, has increasingly been used in health care, often paired with other digital health tools such as wearable sensors, smart home devices, virtual assistants, and robotics [4,5]. For example, wearable sensors combined with AI algorithms can monitor functional status, sleep patterns, and mobility to detect early signs of cognitive or physical decline in community-dwelling older adults [6-8]. AI-enabled robotics with autonomous navigation, multimodal sensing, and assistive manipulation capabilities improve health care efficiency by continuously monitoring patient function, supporting clinical workflows, and reducing preventable hospitalizations for older adults [9-11]. Despite these advancements, the broad adoption of such technologies remains limited.

The literature has examined facilitators of and barriers to general technological adoption by end users, including older adults, care partners, and clinicians [12-15]. These factors, such as perceived usefulness, ease of use, trust, cost, privacy concerns, and social support, are key determinants of technology acceptance in older adult populations [16-19]. The findings have been synthesized in conceptual frameworks that describe the key influences in technology adoption. A commonly used framework is the technology acceptance model (TAM) [20]. The TAM proposes that individuals’ acceptance and use of a technology are primarily determined by 2 key factors: perceived usefulness and perceived ease of use [16]. Subsequent extensions of the TAM have also emphasized additional contextual factors, including trust, cost, privacy, and organizational support, that may influence technology adoption in health care settings [14,16]. Some recent studies have examined attitudes toward and acceptance of technology that can be paired with AI, such as wearable devices and robots [12,14,21,22]. However, we did not find studies that specifically examined how key stakeholders made decisions about AI health technologies designed to improve the care of older adults. We also did not find perspectives on how health system leaders and payers consider technology adoption and how investors and developers consider the decision of what technological products to develop and finance. As health system payers, investors, and developers are responsible for making novel health technologies available, understanding their decision-making in conjunction with that of the end users is critically important.

To address the above-mentioned knowledge gaps, we conducted a qualitative study to explore the perspectives of different stakeholder groups—older adults, care partners, clinicians, payers, investors, and technology developers—regarding their decision-making on the development, financing, and adoption of AI and novel technologies to improve the health of older adults. We aimed to identify areas of consensus and potential differences to help understand how to align stakeholders’ key drivers of decision-making to accelerate the implementation of impactful technologies for the health and well-being of older adults.

## Methods

### Design, Study Setting, and Participant Recruitment

We chose to use qualitative semistructured individual interviews to explore stakeholder perspectives. This project is part of a larger study in which we also explored stakeholder perspectives on priority areas for AI applications, perceived benefits of and concerns about AI, and educational needs; these results have been reported previously [23,24]. In this paper, we report stakeholder perspectives on decision-making on the development, financing, and adoption of AI applications in the care of older adults. This study focused on stakeholders, including older adults (defined as those aged 60 years or older) and care partners for older adults (via self-report); clinicians; health system or health insurance leaders involved in technology adoption, such as the chief information officer (hereafter referred to as “payers”); and investors and technology developers. We combined older adults and care partners in the same group as many older adults were or had been care partners.

This project was supported by the Johns Hopkins Artificial Intelligence and Technology Collaboratory (JHU-AITC) for Aging Research [25]. The Stakeholder Engagement Core within the JHU-AITC engages longitudinally with a stakeholder council of older adults, care partners, and clinicians from Maryland and Iowa. We recruited older adults, care partners, and clinicians via referrals from the study team and council members; we recruited payers, investors, and technology developers via referrals from the study team, JHU-AITC colleagues, and the coordinating center that supports artificial intelligence and technology collaboratories. We also used snowball sampling to recruit contacts of existing participants. Recruitment continued until information saturation was reached.

All participants needed to be English speaking and be able to provide informed consent. Each participant was offered a US $50 gift card.

### Interview Guide

The interview guide was developed by the study team, which included expertise from engineering, business development, patient engagement, geriatric medicine, and social and behavioral science. We defined AI as “technologies where machines try to do what human beings do” and described that there were many types of AI applications, such as alerts, sensors, prediction algorithms, or robots. For older adults, care partners, clinicians, and payers, we asked how they decided whether to use an AI product; for investors, we asked how they decided to invest in an AI product; for developers, we asked how they made decisions on what type of AI product to work on or move forward with in development. The full interview guide is available in Multimedia Appendix 1.

### Data Collection and Analysis

Three team members (NLS, KC, and JM) conducted interviews in person (at private workspaces) or virtually from May 2023 to January 2024. All 3 team members had prior qualitative research experience. One team member (NLS) had prior working relationships with some of the clinician participants.

Interviews were audio recorded and transcribed verbatim; the average duration of the interviews was 34 (SD 11) minutes. We collected demographic information from all participants. For older adults and care partners, we collected information about education, financial strain, health literacy, self-rated health, and duration of caregiving. For clinicians, we asked about their practice setting. For all other participants, we asked about their professional roles and the duration working in that role.

We analyzed interview transcripts using reflexive thematic analysis informed by the approach by Braun and Clarke [26] to identifying, analyzing, and reporting patterns of meaning across qualitative data. This method was appropriate because our study aimed to examine how different stakeholder groups described decision-making on the development, financing, and adoption of AI health technologies for older adults. Consistent with the guidance from Braun and Clarke [26] on good practice in thematic analysis, we used an iterative analytic process that emphasized methodological clarity; reflexive engagement with the data; and alignment among the research questions, analytic procedures, and resulting themes.

To code the transcripts, we used thematic analysis with an integrated approach that used both inductive coding and a deductive organizing framework that drew conceptually from the TAM [16,20]. Specifically, a codebook was developed based on the interview guide and revised iteratively to add new codes generated inductively based on the review of 9 randomly selected transcripts. We organized the codes, when applicable, by domains in the TAM and compared our findings to the TAM during the interpretation of the results. Each transcript was independently coded using the finalized codebook by at least 2 investigators (NLS, KC, and JM), and differences were reconciled through consensus. The investigators involved in the analysis had training in clinical general or geriatric medicine, health service research, and/or qualitative research. Results were summarized into major themes and illustrated via representative quotes. Analysis used the ATLAS.ti software (version 24; ATLAS.ti Scientific Software Development GmbH).

Reflexivity was addressed throughout data collection and analysis. The study team included members with expertise in engineering, business development, patient engagement, geriatric medicine, and social and behavioral science, which allowed for multiple disciplinary perspectives to inform interpretation. At the same time, our team recognized that these professional backgrounds, as well as prior relationships with some participants, could shape data collection and interpretation. To address this, the team discussed emerging interpretations, considered alternative explanations, and examined how researchers’ assumptions and disciplinary perspectives may have influenced theme development.

Several strategies were used to enhance trustworthiness. Credibility was supported through independent coding by multiple investigators and consensus discussions using transcripts. Dependability was strengthened by maintaining documentation of analytic decisions, including codebook revisions, coding discussions, and theme refinement. Confirmability was supported through team-based reflexive discussion and by grounding interpretations in the data rather than a priori assumptions. Transferability was supported by describing the study sample, stakeholder groups, recruitment approach, interview procedures, and analytic process in sufficient detail to allow readers to assess the relevance of the findings to other settings and populations.

### Ethical Considerations

This project was approved by the Johns Hopkins Medicine Institutional Review Board (IRB00356437). The reporting of this study followed the COREQ (Consolidated Criteria for Reporting Qualitative Research) reporting guidelines [27]. Consent to participate was obtained for this project, and it adheres to human ethics guidelines. This study used deidentified data and posed minimal risk to participants.

## Results

### Participants

The 49 participants included 15 (30.6%) older adults or care partners, 15 (30.6%) clinicians, 8 (16.3%) payers, 5 (10.2%) investors, and 6 (12.2%) technology developers (Table 1). The older adult group had a mean age of 71.3 years; 60% (9/15) of the older adults reported caregiving experience. Clinicians comprised physicians, nurses, pharmacists, rehabilitation therapists, and a dentist. Most of the older adult or care partner participants (14/15, 93.3%) and all the clinician participants (15/15, 100%) were from Maryland and Iowa, whereas payer, investor, and technology developer participants were from 9 different states.

**Table 1:** Participant characteristics.

			Older adults and care partners (n=15)		Clinicians (n=15)		Payers (n=8)		Investors and technology developers (n=11)
Age (y), mean (range), SD			71.3 (8.6; 65-93)		50.3 (11.3; 33-69)		51.6 (10.9; 36-65)		42.3 (16.7; 27-62)
Women, n (%)			11 (73.3)		7 (46.7)		3 (37.5)		0 (0)
**Race, n (%)**									
	Asian	1 (6.7)		3 (20)		0 (0)		6 (54.5)	
	Black	3 (20)		0 (0)		0 (0)		1 (9.1)	
	Hispanic	0 (0)		1 (6.7)		0 (0)		0 (0)	
	White	11 (73.3)		11 (73.3)		8 (100)		4 (36.4)	
**Location, n (%)**									
	Maryland	10 (66.7)		8 (53.3)		2 (25)		3 (27.3)	
	Iowa	4 (26.7)		7 (46.7)		2 (25)		0 (0)	
	Other states	1 (6.7)^a^		0 (0)		4 (50)^b^		8 (72.7)^c^	
**Past or current care partner, n (%)**			9 (60)		—^d^		—		—
	Duration as care partner (y), mean (range)	9.5 (0.5-25)		—		—		—	
**Educational level, n (%)**									
	College degree or higher	3 (20)		—		—		—	
	High school or <4-y college	11 (73.3)		—		—		—	
	Missing data	1 (6.7)		—		—		—	
Self-reported financial strain^e^, n (%)			3 (20)		—		—		—
Low health literacy^f^, n (%)			3 (20)		—		—		—
**Self-rated health, n (%)**									
	Excellent or very good	6 (40)		—		—		—	
	Good	4 (26.7)		—		—		—	
	Fair or poor	4 (26.7)		—		—		—	
	Missing	1 (6.7)		—		—		—	
**Clinician practice setting (could choose more than one), n (%)**									
	Inpatient	—		10 (66.7)		—		—	
	Outpatient	—		5 (33.3)		—		—	
	Nursing home	—		3 (20)		—		—	
	Home-based care	—		4 (26.7)		—		—	
Duration in profession (y), mean (SD)			—		19.3 (11.1)		13.3 (9.5)		9.9 (8.5)

^a^Washington, DC.

^b^Colorado, Florida, Massachusetts, and Minnesota.

^c^California, Massachusetts, Virginia, and Washington, DC.

^d^Not asked for these participants.

^e^Financial strain was assessed using the following question: “During the last year, were there any times when you did not have enough money to pay rent or utilities or medical bills?” The response “yes” was counted as having self-reported financial strain [20].

^f^Low health literacy was assessed using the following question: “How confident are you in filling out medical forms by yourself?” Responses of “somewhat,” “a little bit,” and “not at all” were counted as low health literacy [14].

### Themes

Three themes emerged from the interviews. First, stakeholders across groups identified cost, usability, and value as key considerations when making decisions about AI health technologies for older adults, although the meaning of these concepts differed by stakeholder group. Second, these differing interpretations contributed to tensions between end users (older adults, care partners, and clinicians) and those responsible for developing and financing technologies (investors and developers), particularly regarding affordability and the design process. Third, participants described several potential strategies to better align stakeholder priorities and support the adoption of AI technologies for older adults. We describe each of the 3 themes below in detail.

### Theme 1: The Value of AI Meant Different Things to Different Stakeholders

#### Overview

We found that all key stakeholders viewed cost, added value, and usability as important when considering AI health technologies for older adults, but each stakeholder group had different considerations within each concept. Although cost, value, and usability have been previously described as important to technology adoption in the literature, here, we contextualize each by stakeholder group and highlight that the same words encompass vastly different considerations (Table 2).

**Table 2:** Perspectives across stakeholder groups on cost, value, and usability when making decisions about AI health technologies for older adults.

			Consideration		Illustrative quote
**Cost and value**					
	Older adults and care partners	Patient’s out-of-pocket cost		“Price would be a barrier.” [Older adult 5]	
	Clinicians	Patient’s out-of-pocket cost		“Many older adults are on fixed incomes.... The group of older adults that probably need what AI might be able to deliver are just not going to have finance to do that.” [Clinician 1]	
	Payers	Health system cost for purchasing, integrating, and maintaining the technology relative to health system savings from improvement in clinical outcomes		“...what are the things that improve quality outcomes and decrease cost.” [Payer 2]	
	Investors and technology developers	Return on investment		“Bluntly, can you make money off of this?” [Investor 1]	
**Usability**					
	Older adults and care partners	Ease of use for older adults		“It has to be user-friendly. It has to be simple...accommodate slow fingers, eyes that don’t see so well and ears that don’t hear so well.” [Older adult 4]	
	Clinicians	Ease of use for older adults and for clinicians		“If it’s too complex then it makes it harder for our staff to use it and it becomes cumbersome.” [Clinician 9]	
	Payers	Compatibility with existing health system		“It’s the integration into the workflow and the health system that’s important.... We were looking at a product that...I said, ‘come back to us when [it’ll] exist inside our electronic health record.’ Because it just doesn’t work well with the workflow.” [Payer 8]	
	Investors and technology developers	Usability needs across other stakeholder groups		“You have to think like a doctor...about the healthcare need and impact of your project...then there are the technical and engineering type of things you have to think about...there’s also the business related things...commercialization, finance, competition...and the actual implementation.” [Technology developer 5]	

#### Subtheme 1a: Cost and Value

Older adults, care partners, and clinicians made similar comments focused on the patients’ out-of-pocket costs, and most were concerned about affordability. However, some commented on being willing to take on the cost if the technology offered sufficient value; one older adult said the following:

What is the return on investment? I’d rather go without some food if this type of technology could help me.

Older adult 1

Payers considered cost and value as a balance among the upfront cost of the technology to the health system or insurance plan, the expected improvement in clinical outcomes, and the potential savings from those outcome improvements. As such, payers commented that they were more likely to invest in technology that targeted high-cost or high-frequency health events. One payer said the following:

Rare event, but very detrimental and/or costly to the health system...something we really want to try to avoid, [we’d] make the investment there. Something that maybe doesn’t have on an individual basis a lot of impact but is very common like hypertension, [we’d] make the investment there.

Payer 8

Payers also considered indirect costs required for supporting the technology:

If I have technology that we’ve acquired that I then need to hire an employee to interface with. Even though [the technology] is reimbursed, do I get enough reimbursement to hire the people? People are expensive, so I really need a massive return on investment to justify it.

Payer 8

Investors and technology developers considered cost and value as the magnitude of return on investment, which in turn included consideration of the potential market size for a given technology and whether the revenue stream for the technology would be directly from consumers or from insurance reimbursement. One investor said the following:

Who’s going to pay for this and is the market large enough?

Investor 1

#### Subtheme 1b: Usability

Similarly, all stakeholder groups agreed on the importance of technology being easy to use, but they had distinct ideas on what that meant (Table 2). Older adults and care partners commented on technology being user-friendly and adapted to the needs of older adults who may have physical, sensory, and/or cognitive impairments. One older adult said the following:

Artificial intelligence can do many things, but it must be as simple as possible, and it must be user-friendly. If there’s an awful lot that has to be learned by the person dealing with the artificial intelligence, it defeats its purpose.

Older adult 4

Clinicians considered the technology’s user-friendliness to patients as well as to clinicians, especially in light of the existing clinical demands. One clinician said the following:

It has to be really user-friendly for me to be able to use it.

Clinician 9

Another commented that “it has to be simple enough” (clinician 2), especially for clinicians who may already be burned out. Payers also commented on user-friendliness for clinicians but were more concerned about the ease of integration with existing systems. Finally, investors and developers commented on the complexity of trying to understand each stakeholder group’s needs and expectations. One investor talked about an AI technology that was designed to help clinicians generate message responses to patients:

...it’s like a quagmire of validation [with each group].... We talk to physicians...we talk to their staff...we talk to patients, do they like receiving automated AI response?... Then there’s IT security issue.... We have to work with [the electronic health record vendor]..., and we are talking to payers to understand...if we were to bill for this, are you going to accept charges of an AI.

Investor 1

### Theme 2: Tensions in Decision-Making Priorities

**Figure 1:**
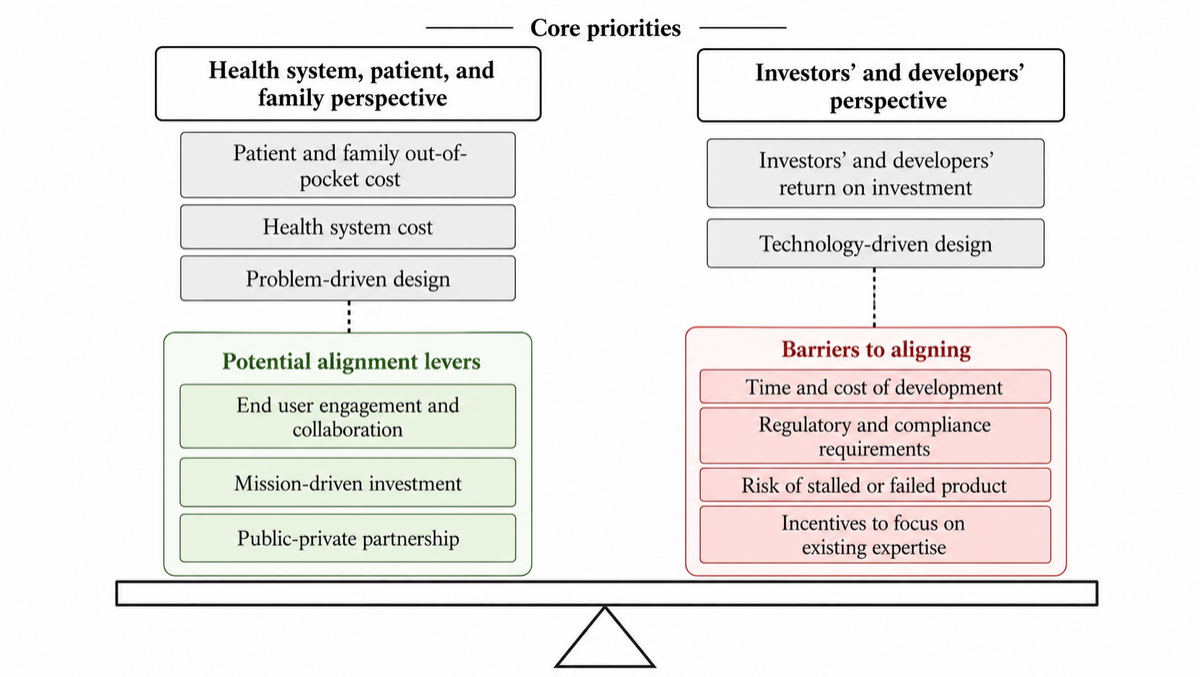
Two identified tension areas between the decision-making priorities of end users and investors and developers were related to cost considerations and the design process. Suggested facilitators of aligning priorities are depicted in green, and existing barriers to aligning priorities are depicted in red.

We observed tension between the decision-making priorities of the end users (eg, older adults, care partners, and clinicians) and those of the developers and investors. The decisional factors that were important to end users were at times in conflict with those that were important to investors and developers. Two areas of tension included the affordability of the technology and the design process (Figure 1).

As stated above, older adults, care partners, and clinicians valued low cost (ie, “costs should be kept as minimal as possible” [older adult 3]). On the other hand, investors and developers commented on multiple considerations in the development process that drive up cost due to the significant risk and the long time horizon involved in developing AI health technologies. Both investors and developers commented that direct-to-consumer products are much higher risk:

...consumer investments are very high risk, especially at early stages.

Investor 4

However, the alternative involves more regulatory cost and time:

...if you decide to go through the prescription route [where] you hope it becomes insured. The cost just balloons, it can be two, three, ten times more expensive...because the [medical device] industry is so conservative...you have to go through a lot of red tape to get it to market.... You are looking at 4 or 5 years to get from napkin sketch to through the FDA.

Investor 4

A developer commented the following on a recent publication looking at novel medical devices:

...after going through the FDA, which [is] very difficult, on average they had to wait 7 years for insurers to cover their technologies...and 44% didn’t even get coverage at all.

Technology developer 5

The investment risks and the long wait, in turn, drive up cost:

...when you have odds like that for novel technologies...investors are going to ask for a big return because the risk is so high. You can’t wait 10 years for a return on something without [the profit] being massive.... There are these huge pressures that drive cost up.

Technology developer 5

A second area of tension was regarding the design process for novel technologies. All stakeholder groups agreed that AI health applications should be designed after first understanding the target users and the health problem. However, many commented that, often, they saw the reverse—that existing technologies were offered to older adults even when they were not the most suitable solutions. One older adult said the following about health technology:

[It feels like] they are designing solutions in search of a problem.

Older adult 1

One engineer said the following:

I think the vast majority of engineers are coming from a place where we have a solution already and we think it might apply to this problem. Because that’s by far easier.... It’s very difficult to come from a purely problem-oriented perspective...even though I think that’s ideal.

Technology developer 5

Another AI researcher commented on the incentive structure being a barrier to problem-driven design:

We have incentive structures to improve on what we already have in our areas of expertise, rather than pursuing new problems and things that may not work. For example, the geriatric population is not extremely well studied from an AI perspective.... It’s much more risky for a researcher to start in the geriatric population and try to come up with a problem that fits AI than to take the AI and fit it to the geriatric population.

Technology developer 2

### Theme 3: Promising Trends and Suggestions for Aligning Priorities

#### Subtheme 3a: Public Education About AI Health Technologies

Promising trends and suggestions can help enhance adoption and align decision-making priorities regarding AI health technology for older adults. Participants commented that older adults may not be sufficiently aware of AI health technology to recognize its value and that better promotion and public education are needed. One older adult said the following:

People need to do a much better job in educating the public on what AI really means and what AI can really do.

Older adult 15

An older adult suggested education through health care providers:

When they go into the doctor’s office, they [can] have information about AI, how it can help.

Older adult 10

Another older adult talked about leveraging television shows to change cultural norms:

...a lot of people watch the soaps and regular TV, if those characters are using technology...it’s going to make it more acceptable.

Older adult 4

One payer commented on more patient-friendly descriptions of AI:

The terminology of AI is not a natural, comfortable, or welcoming description for seniors. I think it’s confusing, threatening, and it doesn’t really get to the fact of helping you.... I would strongly recommend [improving] how we describe this new technology.

Payer 4

#### Subtheme 3b: Multi-Stakeholder Engagement and Problem-Driven Design Are Vital

Participants commented on the importance of engagement and collaboration to better understand the needs and expectations across stakeholder groups and approach technology design with a problem-driven mindset (Figure 1). All stakeholder groups commented on the importance of engaging older adults, care partners, and clinicians in the development of novel technologies. Several investors commented that they now used stakeholder engagement as a metric in evaluating potential projects:

...all good startup should be talking to their stakeholders before they even start developing a product...so we [ask]...how many people have you chatted with? What kind of feedback did they give?

Investor 3

Going beyond unilateral engagement from developers to end users, several participants also commented on the importance of collaboration and active participation from the public and clinicians. One payer who was also a clinician advocated for clinicians to be involved in technology development:

I wish more physicians realized the central place that software takes in our care, and realized that you can be just as much a physician designing a [health] application as you can on the wards.

Payer 6

#### Subtheme 3c: Public-Private Partnerships May Reduce Cost Barriers

Finally, participants commented on trends and suggestions to help align cost expectations for new technologies (Figure 1). One investor commented that some investors are willing to accept less profit because they are passionate about older adults and improving their health:

...there are...founders who don’t care about the money part as much, and they are really passionate about a certain idea.... Maybe they had a family member that went through the same thing and they really want to solve this issue.

Investor 3

Another commented on public-private partnerships that can help align incentives:

...the economics may not be there to develop a solution for this...group, but if you can de-risk the research and development costs, [which] is the whole premise of the federal government funding, then it changes the dynamics for commercialization of those ideas.

Investor 1

## Discussion

### Principal Findings

This is the first study, to our knowledge, to examine decision-making related to AI technology development, financing, and adoption across diverse stakeholder groups. While previous research and frameworks such as the TAM have broadly acknowledged that cost, usability, and perceived value are significant determinants of technology acceptance by end users [28], this study adds depth by demonstrating how these concepts hold varied and nuanced meanings among stakeholder groups. In general, older adults and clinicians shared similar perspectives as end users, whereas investors and technology developers shared similar perspectives as those responsible for bringing about innovation; payers tended to have distinct perspectives from those of the other groups as they focused on broader, system-level concerns. The findings on the tension areas between decision-making priorities across stakeholder groups are also novel, drawing attention to factors in the existing technology development process that contribute to competing priorities.

How the different stakeholders viewed cost, value, and usability is consistent with what has been reported regarding other health technologies, but these have usually been reported *separately* and in different fields of study. For example, older adults, care partners, and clinicians primarily interpreted cost in terms of affordability, which has also been shown in studies on patient behavior regarding adoption of health care technologies [17,29]. Payers framed cost and value in terms of return on investment through reduced costs associated with high-frequency or high-severity clinical outcomes, a viewpoint common in the health care economics literature [30,31]. Investors and technology developers interpreted cost and value through the economic lens of market viability and profitability [32]. Our study design offering a juxtaposition of perspectives is a strength. A comparison of stakeholder perspectives has been described in the adoption of other technologies, such as patient portal use, but has focused on implementation without including the perspectives of developers or investors [18,33].

Recognizing that the same words and labels (eg, “cost” or “usability”) represent different concepts to different stakeholders can help entrepreneurs and technology developers be more considerate and specific in the design process to meet stakeholder needs. More specific descriptions of these considerations may help highlight their importance. For example, instead of cost and usability, it may be more helpful for developers to recognize that older adults value products that are affordable and easy to use for individual users, whereas payers value technology that reduces health care costs and is easy to implement within an existing health care system. Similarly, funders of AI health technology research can evaluate proposals with metrics that are inclusive of different stakeholder viewpoints, for example, ensuring that a project addresses both individual usability and ease of health system–level implementation.

AI is unique in that the same underlying product can be used in multiple contexts. For example, large language models can be a direct-to-consumer tool (eg, ChatGPT) or a clinician-facing decision support system (eg, OpenEvidence). In contrast, prior health technologies such as patient portals have much more defined and stable target users. AI’s adaptability means that the target user can shift after a product has already been developed and deployed. This has important implications for stakeholder alignment: the cost, usability requirements, accountability structures, and regulatory pathways may differ substantially depending on the target user. This flexibility creates both opportunity, allowing products to find their highest-value use case, and risk as it can obscure whose needs the technology was actually designed to serve.

Considering multiple perspectives together revealed critical tensions between end users (older adults, care partners, and clinicians) and technology creators (developers and investors). These tensions, particularly regarding affordability and design processes, signal significant barriers to wider adoption of patient-centered, impactful AI technologies for older adults. AI operates within a uniquely heterogeneous regulatory landscape where AI health applications can enter the market through multiple channels [34]. Some can be marketed directly to consumers as wellness products, which are subject to minimal regulatory oversight, whereas others may be considered medical devices that need to meet rigorous requirements before approval by the Food and Drug Administration [35]. Investors and developers highlighted factors in both pathways that contributed to tensions regarding affordability and design of AI health applications, including that direct consumer-facing technologies posed substantial financial risks whereas regulated products faced significant regulatory hurdles and lengthy approval timelines. The related incentive misalignments favor incremental enhancements to existing technologies over exploratory problem-driven innovations addressing complex issues in older populations, which is a phenomenon that has also been reported in more traditional medical device development [35]. Indeed, the current health technology development infrastructure may very well inadvertently motivate high-cost products that are “solutions in search of a problem,” and our results highlight the need for change.

Potential changes may include a revised or new regulatory and approval pathway for AI health technologies that is better tailored to the specific risks involved. Another change may involve altering the incentive structures, at least in research environments with a mission of discovery, to motivate problem-driven design and exploration of new ideas over honing existing expertise. We also identified actionable suggestions to facilitate adoption and align decision-making priorities from participants. Closer engagement across stakeholder groups throughout the development process was suggested by participants as one key solution to problem-driven design, which has also been reported in prior literature [12]. However, for older adults and clinicians to be interested and willing to engage, there first needs to be better public education and awareness campaigns to demystify AI and make clear its potential benefits. Participants suggested specific approaches for educating older adults about AI, such as through health care providers as well as media and cultural norms. Participants also suggested including stakeholder engagement as an explicit criterion for investment or funding, which can enhance accountability. Another suggestion by participants identified financial models such as public-private partnerships to balance cost considerations across stakeholders. The JHU-AITC is an example of one such partnership where public funding is awarded to promising pilot AI projects in very early development to help them advance to a stage where they can successfully attract private investment. Finally, it may be important to learn from other countries and health care models in terms of how they approach stakeholder engagement in AI health application development.

We acknowledge that our study has limitations. First, our participants were only restricted to English speakers, which may limit the generalizability of our findings to non-English speakers. Second, most older adult, caregiver, and clinician participants were based in Maryland and Iowa in the United States, which may limit geographic transferability. However, the inclusion of Iowa residents allowed us to obtain perspectives from rural residents and clinicians, where technology adoption is often delayed or limited. Third, several stakeholder groups lacked gender and racial diversity. For instance, all investor and technology developer participants were male. We did not assess prior experiences and familiarity with AI among our participants. Our future work should build on these findings through larger surveys with more diverse and representative samples across stakeholder groups.

### Conclusions

In sum, we found that, across diverse stakeholder groups, the concepts of cost, usability, and value were considered universally important but had different meanings across different groups. This finding can help inform future research and technology development by highlighting that different elements of cost, usability, and value need to be separately considered and evaluated to meet the needs of diverse stakeholders. We also found competing decision-making priorities among different stakeholder groups when considering AI health applications for older adults, especially between those responsible for making new AI technologies available and the end users of such technologies. Implementing participant suggestions and other strategies to better align these priorities is essential for ensuring the widespread adoption of patient-centered, impactful AI solutions for older adults.

## Appendix

Multimedia Appendix 1 Interview guide.

## Abbreviations

COREQ: Consolidated Criteria for Reporting Qualitative Research
JHU-AITC: Johns Hopkins Artificial Intelligence and Technology Collaboratory
TAM: technology acceptance model
