# Supplementary material for: The Ways in Which Stakeholders Make Decisions About AI and Novel Technologies for the Health Care of Older Adults: Qualitative Interview Study
Source: JMIR Aging. 2026 Jul 29;9:e86148. doi: 10.2196/86148 (PMC13469834; doi:10.2196/86148)
Supplement: Supplementary file 1 [file aging_v9i1e86148_app1.docx]

**Appendix**

**Interview Guide**

Thank you for being part of this study. We would like to understand how to use new technologies to improve the health and well-being of older adults and their caregivers. We are interested specifically in artificial intelligence; Artificial intelligence describes technologies where machines try to do what human beings do. There are many types of AI systems – for example, there are alert or reminder systems to remind people to take medicines, smart watches or wearable sensors that track people’s steps and heart rates, computer algorithms that predict falls based on the data, or robots that provide companionship.

We want to know your opinions about what should be the focus of artificial intelligence, i.e. what problems should artificial intelligence be trying to solve? There is no right or wrong answer.

Older adults/caregivers

1. What are the most important problems or challenges that you experience related to your health or daily function or when you care for your [relationship to patient]? (PROBE: this could be related to physical, mental, or emotional health) How do you currently deal with these problems?
2. [If applicable: The problems you mentioned are the ones you are experiencing now at home,] we would also like to know what problems you may have experienced when you or your [relationship to patient] was in the hospital, in the emergency room, or skilled nursing facility. Can you tell us about any related experiences? How did you deal with those?
3. Specifically about artificial intelligence. What have you heard about AI? What thoughts do you have about AI? What concerns, if any, do you have about AI?
4. We are trying to understand what health-related problems AI should focus on.   We do not expect you to come up with the technology solutions, but if you have any thoughts or suggestions, we’d love to hear them – i.e. are there technologies that you think should be developed to address the problems you mentioned before?
5. What technologies do you already use to help with your (your loved one’s) health? How have these worked? How do these technologies help? What challenges have you had? Do you need help using technology?
6. For health-related issues, are there technologies that you decided against trying? Or used in the past but no longer? Why?
7. What are the top factors you consider when deciding whether or not to use a new AI technology for your health? What are the barriers?
8. What do you want technology developers and researchers to know as they work in this area?

Clinicians

1. Can you please start by sharing a bit about your role in the care of older adults? Such as what setting you practice in and what you do.
2. What are the most important problems or challenges that you experience when caring for older adults? These could be challenges you experience when you care for them or could be what you perceive that the older adults or caregivers are experiencing? (PROBE: this could be related to physical, mental, or emotional health) How do you or they currently handle these problems?
3. [If applicable: The problems you mentioned are the ones experienced in the outpatient or home setting,] we would also like to know what problems you experience in other settings (if you practice there) or that you perceive that older adults or caregivers experience when they are in other settings? How do you or they handle those challenges?
4. Specifically about artificial intelligence. What have you heard about AI? What thoughts do you have about AI? What concerns, if any, do you have about AI?
5. We are trying to understand what problems should AI focus on. We do not expect you to come up with the technology solutions, but if you have any thoughts or suggestions, we’d love to have them – i.e. are there technologies that you think should be developed to address the problems you mentioned before?
6. What technologies do you use in the care of older adults? How have these worked for you? How do these help? What challenges have you experienced? Are there technologies that you stopped using? If so, why?
7. What technologies do you see your patients or caregivers using for health-related issues or daily function?  What technologies, if any, do you currently recommend to your patients or caregivers to use for health-related issues?  How have these worked for your patients? How do these technologies help? What challenges have you seen? What technologies do you think work well for older adults and why?
8. Are there technologies that you recommended your patients not to use? Are there technologies that you have seen your patients stop using? If so, why?”
9. What are the top factors you consider when deciding whether or not to use a new AI technology when caring for patients or recommending your patients to use?  What are the barriers?
10. What do you want technology developers and researchers to know as they work in this area?

Payers

1. Can you share a little bit of your background in your profession, what is your title and what you do?
2. What are the most important problems or challenges that older adults and caregivers experience (in your health system/health plan/facility/organization)? How are these currently managed?
3. [If applicable: The problems you mentioned are the ones experienced in the outpatient or home setting,] we would also like to know what problems older adults or caregivers may be experiencing in the hospital or in the emergency room?
4. Specifically about artificial intelligence. What have you heard about AI? What thoughts do you have about AI? What concerns, if any, do you have about AI?
5. We are trying to understand what problems should AI focus on. We do not expect you to come up with the technology solutions, but if you have any thoughts or suggestions, we’d love to have them – i.e. are there technologies that you think should be developed to address the problems you mentioned before?
6. Can you tell me about the most recent time your organization decided to adopt a new technology for older adults or for clinicians in their care of older adults? What have been the benefits? What about challenges?
7. Have there been times where you decided against adopting a new technology or stopped using a technology? What was that technology and why?
8. What are the top factors you consider when deciding whether or not to adopt/invest in a new AI technology for the care of older adults? What information or data do you need to make that decision? What are the barriers in adopting a new technology?
9. What do you want technology developers and researchers to know as they work in this area?

Investors

1. Can you share a little bit of your background in your profession, what is your title and what you do?
2. Do you invest in AgeTech? Where do you see the most important or exciting opportunities as investors? What problems in older adults’ health and wellbeing can AI or address the best in your opinion? What are some potential applications that you would love to see or are excited about?
3. What thoughts do you have about AI? What are the benefits? What concerns, if any, do you have about AI? What added value or return on investment do you expect AI to add?
4. What are the key opinion leaders you seek when making investments in Age Tech? Do you include older adults or clinicians? Whose voices matter most?
5. Can you tell me about the most recent time you decided to invest in a new AI technology? How was that decision made? What were the key factors for this decision?
6. Can you discuss a time when you discontinued an investment or exited early from a new AI technology. Why? What happened to the tech? What were some lessons you learned from that decision?
7. What do you want technology developers and researchers to know about investors’ priorities as they work in the AgeTech domain?
8. If older adults and caregivers or clinicians identify the most important problem they want AI to help solve, how might we motivate investors to also focus on those areas?

Technology developers

1. Can you share a little bit of your background in your profession, what is your title and what you do?
2. Thinking about AI or new technology development, can you tell me how you select an area to focus on? What are some of the most important or exciting areas or ideas in the field? What are some potential applications that you would love to see or are excited about? What would attract people to focus on older adults? What might turn people off from working on products for older adults?
3. What are your thoughts about AI? What are the benefits? What concerns, if any, do you have about AI?
4. At what point during the development process do you get input or feedback from the intended end users? What kind of questions do you ask to receive feedback?
5. What challenges have you encountered in developing new AI technologies? What factors are considered when deciding whether or not a new technology will move forward in development or not?
6. What challenges have you seen in adoption of new AI technologies? Any specific facilitators or barriers for older adults’ adoption of new technologies?

7. In your opinion, what are some of the most important problems that older adults and caregivers experience? What are the top priority areas in older adults’ health and wellbeing that AI can and should address in your opinion?
8. If older adults and caregivers or clinicians identify the most important problem they want AI technology to help solve, how might we motivate technology developers and researchers to then work on these areas?
